# Contribution of *ARLTS1* Cys148Arg (T442C) Variant with Prostate Cancer Risk and ARLTS1 Function in Prostate Cancer Cells

**DOI:** 10.1371/journal.pone.0026595

**Published:** 2011-10-20

**Authors:** Sanna Siltanen, Tiina Wahlfors, Martin Schindler, Outi R. Saramäki, John Patrick Mpindi, Leena Latonen, Robert L. Vessella, Teuvo L. J. Tammela, Olli Kallioniemi, Tapio Visakorpi, Johanna Schleutker

**Affiliations:** 1 Institute of Biomedical Technology/BioMediTech, University of Tampere and Centre for Laboratory Medicine, Tampere University Hospital, Tampere, Finland; 2 Institute for Molecular Medicine, University of Helsinki, Helsinki, Finland; 3 Department of Urology, University of Washington, Seattle, Washington, United States of America; 4 Puget Sound VA Medical System, Seattle, Washington, United States of America; 5 Department of Urology, University of Tampere and Tampere University Hospital, Tampere, Finland; 6 Department of Medical Biochemistry and Genetics, University of Turku, Turku, Finland; University of Nebraska Medical Center, United States of America

## Abstract

*ARLTS1* is a recently characterized tumor suppressor gene at 13q14.3, a region frequently deleted in both sporadic and hereditary prostate cancer (PCa). *ARLTS1* variants, especially Cys148Arg (T442C), increase susceptibility to different cancers, including PCa. In this study the role of Cys148Arg substitution was investigated as a risk factor for PCa using both genetic and functional analysis. Cys148Arg genotypes and expression of the *ARLTS1* were explored in a large set of familial and unselected PCa cases, clinical tumor samples, xenografts, prostate cancer cell lines and benign prostatic hyperplasia (BPH) samples. The frequency of the variant genotype CC was significantly higher in familial (OR = 1.67, 95% CI = 1.08–2.56, *P* = 0.019) and unselected patients (OR = 1.52, 95% CI = 1.18–1.97, *P* = 0.001) and the overall risk was increased (OR = 1.54, 95% CI = 1.20–1.98, *P* = 0.0007). Additional analysis with clinicopathological data revealed an association with an aggressive disease (OR = 1.28, 95% CI = 1.05-∞, *P* = 0.02). The CC genotype of the Cys148Arg variant was also contributing to the lowered *ARLTS1* expression status in lymphoblastoid cells from familial patients. In addition significantly lowered *ARLTS1* expression was observed in clinical tumor samples compared to BPH samples (*P* = 0.01). The *ARLTS1* co-expression signature based on previously published microarray data was generated from 1587 cancer samples confirming the low expression of ARLTS1 in PCa and showed that *ARLTS1* expression was strongly associated with immune processes. This study provides strong confirmation of the important role of *ARLTS1* Cys148Arg variant as a contributor in PCa predisposition and a potential marker for aggressive disease outcome.

## Introduction

ADP-ribosylation factor-like tumor suppressor gene 1 (*ARLTS1*), also known as ADP-ribosylation factor-like protein 11 (*ARL11*), is a newly characterized gene located at locus 13q14.3. ARLTS1 belongs to the ADP-ribosylation factor (ARF)-ARF-like (ARL) family of the Ras protein superfamily [1]. ARFs are guanine-nucleotide-binding proteins which are critical components of several different eukaryotic vesicle trafficking pathways. As with other members of the Ras superfamily, ARFs function as molecular switches by cycling between inactive GDP- and active GTP-bound conformations [2]. ARLTS1 has been characterized as an intracellular protein having tissue specific expression in the lung and leucocytes. *ARLTS1* variants, such as the nonsense polymorphism Trp149Stop (G446A) and missense polymorphism Cys148Arg (T442C), have been suggested to have a role in different cancers [3]–[6].

Prostate cancer is the most frequently diagnosed cancer in males in many countries, including Finland. Aging and improved diagnostics most evidently increase the number of new cases, but the incidence is influenced also by some unknown factors. Growing number of new cases create pressure to health care system and new tools for PCa diagnostics, prognostics and treatment are required, especially to avoid over treatment and unnecessary biopsies. During the last several years there has been extensive research in PCa etiology and genome-wide association studies have revealed several common low penetrance genetic alterations. The association of these variants with clinicopathologic features and prognosis remains unclear and results are lacking clinical implications.

We recently showed a significant association withCys148Arg (T442C) variant and the risk of PCa [7]. Further evaluation of this variant is warranted to increase the power of the association and study the functional role of the variant in PCa. More samples are also needed to evaluate the implication of this variant to clinical outcome and a potential role in predictive biomarker of PCa.

Besides the genetic variants, DNA copy number aberrations are one of the most frequently observed genetic changes in familial and sporadic PCa [8]–[10]. In most of the cases target genes for the aberrations are not fully identified. Interestingly, allelic imbalance (AI) has been detected at 13q14.2-13q14.3, and it is an important event in the progression of localized PCa [11]. Differences of 13q14 loss of heterozygosity (LOH) in different PCa groups could also be used to distinguish clinically insignificant PCa [12], [13].

In this study we analyzed the role of *ARLTS1* in more detail, especially the role of Cys148Arg (T442C) in PCa risk. Chromosomal aberration in 13q14.3 was analyzed with aCGH to evaluate the *ARLTS1* copy number changes in PCa xenografts and cell lines. The expression of *ARLTS1* was studied in clinical tumor samples, BPH samples and also co-expression data form previously published data was analyzed.

## Methods

### Study population

All samples collected are of Finnish origin. Identification and collection of the Finnish HPC families have been described elsewhere [14]. The familial samples genotyped in this study had at least one affected first or second degree relative. The clinical characteristics of the familial patients can be found in Table S1.

The unselected consecutive prostate cancer patients were diagnosed with PCa between 1999 and 2005 in the Department of Urology at Tampere University Hospital. The hospital is a regional referral center in the area for all patients with PCa, which results in an unselected, population-based collection of patients. The clinical characteristics of the consecutive unselected PCa can be found in Table S1.

A set of benign prostatic hyperplasia (BPH) cases was also used in this study. The diagnosis of this BPH cohort was based on lower-urinary tract symptoms, free uroflowmetry, and evidence of increased prostate size obtained by palpation or transrectal ultrasound. If PSA was elevated or digital rectal examination or transrectal ultrasound showed any abnormality indicative of PCa, the patients underwent biopsies to exclude diagnoses of PCa, high-grade prostate intraepithelial neoplasia (PIN), atypical small acinar cell proliferation (ASAP), or suspicion of malignancy.

Clinical prostate tumors were obtained from Tampere University Hospital (Tampere, Finland) including freshly frozen prostate tumor specimens representing benign prostate hyperplasia (BPH, *n* = 14), androgen-dependent (*n* = 14) and hormone-refractory (*n* = 6) carcinomas. The specimens were histologically examined for the presence of tumor cells using H&E staining. Only samples containing >60% cancerous or hyperplastic epithelial cells were selected for the analyses. The BPH samples were obtained from prostatectomy specimens from cancer patients and were histologically verified not to contain any cancerous cells. Samples from hormone-refractory carcinomas were obtained from transurethral resections of prostate (TURP) from patients experiencing urethral obstruction despite ongoing hormonal therapy. The time from the beginning of hormonal therapy to progression (TURP) varied from 15 to 60 months. The use of clinical tumor material was approved by the Ethical Committee of Tampere University Hospital.

The control samples consisted of DNA samples from anonymous, voluntary, and healthy blood donors obtained from the Blood Center of the Finnish Red Cross in Tampere. The EDTA blood samples used as reference in Q-RT-PCR were from healthy anonymous donors.

Patient information and samples were obtained with full informed consent. The study was performed under appropriate research permissions from the Ethics Committees of the Tampere University Hospital, Finland, as well as the Ministry of Social Affairs and Health in Finland.

### Cell lines and xenografts

The PCa cell lines LNCaP, DU145, PC-3, NCI-660 and 22Rv1 were obtained from American Type Culture Collection (Manassas, VA, USA), whereas LAPC-4 was kindly provided by Dr. Charles Sawyers (UCLA, Los Angeles, CA), and the VCaP cell line was provided by Dr. Jack Schalken (Radboud University Nijmegen Medical Centre, Nijmegen, the Netherlands). All cell lines were cultured under recommended conditions. PrEC primary cells were obtained from Lonza (Walkersville, MD, USA). EP156T is an h-tert immortalized normal prostate epithelial cell line [15] that was made available by one of the authors (O.K.). DNA was extracted according to standard laboratory protocols and total RNA was extracted with the TRIzol reagent (Invitrogen Life Technologies, Carlsbad, CA, USA).

Nineteen human PCa xenografts of the LuCaP series used in direct sequencing and fifteen of them used in Q-RT-PCR were made available by one of the authors (R.L.V.) and have been described elsewhere [16], [17].

The lymphoblastoid cell lines were derived by Epstein-Barr virus transformation of peripheral mononuclear leucocytes from patients. Lymphoblastoid cell lines were grown in RPMI-1640 medium (Lonza, Walkersville, MD, USA) supplemented with 10% fetal bovine serum (Sigma-Aldrich, St. Louis, MO, USA) and antibiotics. Cell pellets were snap-frozen and total RNA was extracted from cells with Trizol® according to the instructions of the manufacturer (Invitrogen, Carlsbad, CA, USA).

### Mutation screening

Mutation screening of the genomic DNA was performed by direct sequencing. Sequencing was performed in an Applied Biosystems 3130xl Genetic Analyzer (Life Technologies Corporation, Carlsbad, CA, USA) according to the instructions of the manufacturer. Primers and PCR conditions used in the mutation screening are available upon request.

### Genotyping

Genotyping was done with the Custom TaqMan® SNP Genotyping Assay using the ABI Prism 7900HT sequence detection system (Life Technologies Corporation, Carlsbad, CA, USA) according to the manufacturer's instructions. Primers and probes for the Cys148Arg (T442C) SNP rs3803185 were supplied by Applied Biosystems via the File Builder 3.1 software.

### Quantitative real-time reverse transcription-PCR

Gene expression analyses were done using LightCycler® (Roche, Mannheim, Germany) and Bio-Rad CFX96™ Real-Time PCR detection system (Bio-Rad Laboratories, Hercules, CA, USA). Total RNA was isolated from cell lines, clinical tumor samples and xenografts as described previously [18], [19]. RNA from cell lines was reverse transcribed into first-strand cDNA using SuperScript™ III First-Strand Synthesis SuperMix kit (Invitrogen, Carlsbad, CA, USA) and random hexamers. RNA from clinical tumor samples and xenografts was reverse transcribed as described previously [18], [19]. Normal human prostate RNA was obtained from Ambion (Cambridgeshire, United Kingdom). For the LightCycler® analyses, primers and probe sets were obtained from TIB MolBiol (Berlin, Germany). The PCR reactions were performed with a LightCycler® FastStart DNA Master^PLUS^ HybProbe kit (Roche, Mannheim, Germany). LightCycler® software (Roche, Mannheim, Germany) was used for data analysis. The TaqMan assay for *ARLTS1* was used for Bio-Rad analyses. The primers and probes were obtained from TIB MolBiol (Berlin, Germany). Bio-Rad's iQ Supermix was used for the PCR reactions and CFX Manager Software™ version 1.6 for data analysis. Expression levels of *ARLTS1* were normalized against the housekeeping gene *TBP* (TATA box binding protein) or against RNA levels. *TBP* was chosen as the reference gene, because there are no known retropseudogenes for it, and the expression of *TBP* is lower than that of many commonly used, abundantly expressed reference genes [20].

### Western blotting

Cells were lysed in a buffer containing 50 mM Tris-HCl pH 7.5, 150 mM NaCl, 0.5% Triton-X-100, 1 mM DTT, 1 mM PMSF and 1× complete protease inhibitor cocktail (Roche, Mannheim, Germany). Lysates were sonicated 4×30 s with Bioruptor instrument (Diagenode, Liege, Belgium) and the cellular debris was removed by centrifugation. Proteins were separated by polyacrylamide gel electrophoresis (SDS-PAGE) and transferred to PVDF membrane (Immobilon-P; Millipore, Billerica, MA, USA). Primary antibodies used were anti-ARL11 (Aviva Systems Biology, San Diego, CA, USA) and anti-actin (pan, clone ACTN05, Neomarkers, Fremont, CA, USA), which were detected by HRP-conjugated secondary antibodies (DAKO, Denmark) and Western blotting luminol reagent (Santa Cruz Biotechnologies, CA, USA) by autoradiography.

### Array comparative genomic hybridization

Array comparative genomic hybridization (aCGH) was performed on PCa cell lines and xenografts with the Human Genome CGH Microarray Kit 244A (Agilent, Santa Clara, CA, USA), and as described in Saramäki OR et al, 2006 [19].

### Statistical analysis

Association of the Cys148Arg (T442C) variant was tested by logistic regression analysis using SPSS statistical software package (SPSS 15.0; SPSS Inc, Chicago, IL, USA). Association with clinical and pathological features of the disease (age at onset, PSA value at diagnosis, T, N, and M-stage, WHO grade and Gleason score) was tested among familial and unselected PCa cases by the Kruskal–Wallis test, Fisher's exact test, and t-test included in R “Stats” package. PCa patients were classified as having an aggressive disease if they had any of the following characteristics: a locally advanced or metastatic tumor (stage T3, T4, N1, or M1, based on pathology if radical prostatectomy was done; otherwise, clinical stage), tumor Gleason grade at diagnosis ≥7, poorly differentiated grade (WHO grade III, if no Gleason grade available) or pretreatment PSA at diagnosis ≥20 ng/ml.

### Co-expression analysis of *ARLTS1* in previously published microarray datasets

The *ARLTS1* co-expression signature was generated from the GeneSapiens [21] database of mRNA expression. *ARLTS1* expression among 1587 tumor samples and among 497 normal samples was determined. We used Pearson correlation to identify genes that positively and negatively correlated with *ARLTS1* among normal samples and tumor samples.

### Functional annotation analysis

Gene sets were explored for enrichment of functional annotation categories such as gene ontology (GO), using tools within the EASE program (http://david.abcc.ncifcrf.gov/) [22]. The top 100 genes that were positively correlating with *ARLTS1* among normal samples were checked for enriched Gene Ontology.

## Results

The whole coding region of *ARLTS1* was previously screened in a total of 164 familial PCa patients and in a total of 377 unselected PCa patients, as well as in 381 control samples [7]. Here the Cys148Arg (T442C) variant was further genotyped in 48 familial PCa patients, 1471 unselected PCa patients, 375 BPH patients and 379 controls. By utilizing our previous data and genotyping more familial and unselected PCa cases, we validated a statistically significant association between Cys148Arg (T442C) and PCa risk (Table 1). When considering the familial PCa cases, and the association with risk allele C, the homozygous form CC reached a *P* value 0.019 (OR 1.67; 95% CI 1.08–2.56). Within unselected cases, the risk was also significant (OR 1.52, 95% CI 1.18–1.97, *P* = 0.001). When combining the datasets of familial and unselected cases (2060 samples total), the Cys148Arg (T442C) variant was found to be associated significantly with PCa risk (OR 1.54, 95% CI 1.20–1.98, *P* = 0.0007). Significant association was only found when comparing the frequencies of the homozygous form of the risk allele C to the frequencies of the homozygous form of the wild-type allele T. No association was found between the frequency of CC and BPH (Table 1).

**Table 1:** Association of the *ARLTS1* Cys148Arg variant with prostate cancer and benign prostatic hyperplasia.

	Cases	Controls			
	n (%)	n (%)	OR*	95% CI	*P*
**Prostate cancer patients, all**	2060	760			
*TT*	615 (29.9)	249 (32.8)			
*TC*	1008 (48.9)	396 (52.1)	1.03	0.85–1.24	0.753
*CC*	437 (21.2)	115 (15.1)	1.54	1.20–1.98	0.0007
*TC+CC*	1445 (70.1)	511 (67.2)	1.15	0.96–1.37	0.137
**Familial prostate cancer patients**	212	760			
*TT*	65 (30.7)	249 (32.8)			
*TC*	97 (45.7)	396 (52.1)	0.94	0.66–1.33	0.723
*CC*	50 (23.6)	115 (15.1)	1.67	1.08–2.56	0.019
*TC+CC*	147 (69.3)	511 (67.2)	1.1	0.79–1.53	0.563
**Unselected prostate cancer patients**	1848	760			
*TT*	550 (29.8)	249 (32.8)			
*TC*	911 (49.3)	396 (52.1)	1.04	0.86–1.26	0.676
*CC*	387 (20.9)	115 (15.1)	1.52	1.18–1.97	0.001
*TC+CC*	1298 (70.2)	511 (67.2)	1.15	0.96–1.38	0.131
**Benign prostatic hyperplasia**	375	760			
*TT*	110 (29.3)	249 (32.8)			
*TC*	203 (54.1)	396 (52.1)	1.16	0.88–1.54	0.299
*CC*	62 (16.5)	115 (15.1)	1.22	0.83–1.79	0.306
*TC+CC*	265 (70.7)	511 (67.2)	1.17	0.90–1.54	0.243

*compared to TT homozygotes.

**Figure 1:**
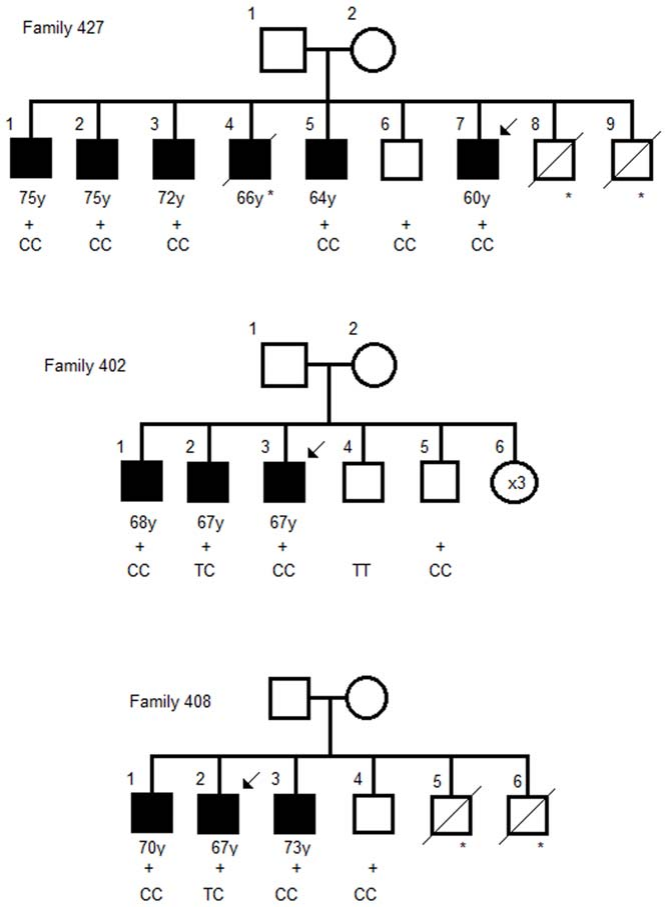
Examples of segregation analysis in three *ARLTS1* Cys148Arg (T442C) variant-positive families with prostate cancer. Squares represent males; circles represent females. Open symbols indicate no neoplasm, and filled symbols denote prostate cancer cases. +indicates the presence of the T442C variant in the DNA sample of the family members, followed by the actual genotype. An arrow indicates the individual initially screened for *ARLTS1* sequence variants. Age at diagnosis for prostate cancer patients (in years) is indicated below the symbol for each family member. An asterisk (*) denotes the persons with no sample available.

When studying the segregation of Cys148Arg (T442C) CC genotype in 86 families where the index was detected as a carrier, complete segregation of the variant CC with cancer phenotype was seen in one family (family 427 in Fig. 1). In the rest of the families segregation was incomplete, yet indicating a clear association with *ARLTS1* genotype CC or TC and cancer phenotype (families 402 and 408 in Fig. 1).

Direct sequencing was used to examine the frequencies of *ARLTS1* variants in clinical prostate tumors. In the clinical prostate carcinomas, we found variants at four sites, Gly65Val (G194T), Pro131Leu (C392T), Cys148Arg (T442C) and Trp149Stop (G446A) (Table 2). At the Cys148Arg (T442C) site, the frequency of the cancer associated risk genotype CC was relatively high (28.3%) compared to the combined frequency of familial and unselected cases 21.2% (Table 1). However, the highest frequency of Cys148Arg (T442C) CC genotype was detected in xenograft samples (42.1%). This is intriguing since LuCaP xenograft samples are considered as a very homogenous representation of PCa.

**Table 2:** Observed *ARLTS1* variants and their frequencies in clinical prostate tumors and LuCap xenografts.

Nucleotide change	Amino acid change	Genotype	Clinical prostate tumors n (out of 53, %)	Xenografts n (out of 19, %)
G194T	Gly65Val	GG	52 (98.1)	19 (100)
		GT	1 (1.9)	0 (0)
		TT	0 (0)	0 (0)
C392T	Pro131Leu	CC	43 (81.1)	19 (100)
		CT	10 (18.9)	0 (0)
		TT	0 (0)	0 (0)
T442C	Cys148Arg	TT	23 (43.4)	7 (36.8)
		TC	15 (28.3)	4 (21.1)
		CC	15 (28.3)	8 (42.1)
G446A	Trp149Stop	GG	52 (98.1)	18 (94.7)
		GA	1 (1.9)	1 (5.3)
		AA	0 (0)	0 (0)

**Figure 2:**
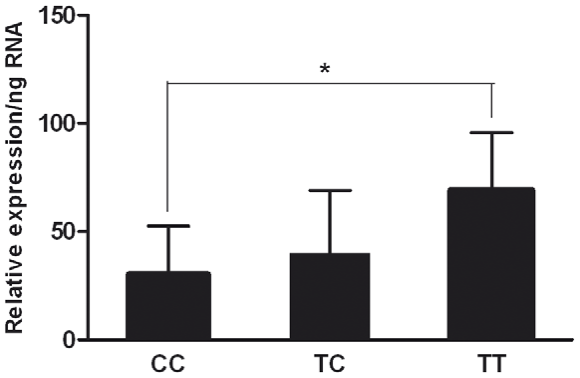
The relative expression of *ARLTS1* in Cys148Arg (T442C) genotyped lymphoblastoid cell lines. Determined by Q-RT-PCR. *, *P*<0.05. Columns, mean of eight individuals; bars, SD.

To investigate the role of Cys148Arg (T442C) genotype in *ARLTS1* expression we selected 24 familial patients, including eight from each genotype group TT, CT and CC. Expression analyses by Q-RT-PCR were carried out by using the RNA extracted from lymphoblastoid cell lines of the patients. *ARLTS1* mRNA was expressed differentially between the three genotype groups (Fig. 2). The expression was significantly reduced among patients carrying the CC genotype (*P* = 0.02).

**Figure 3:**
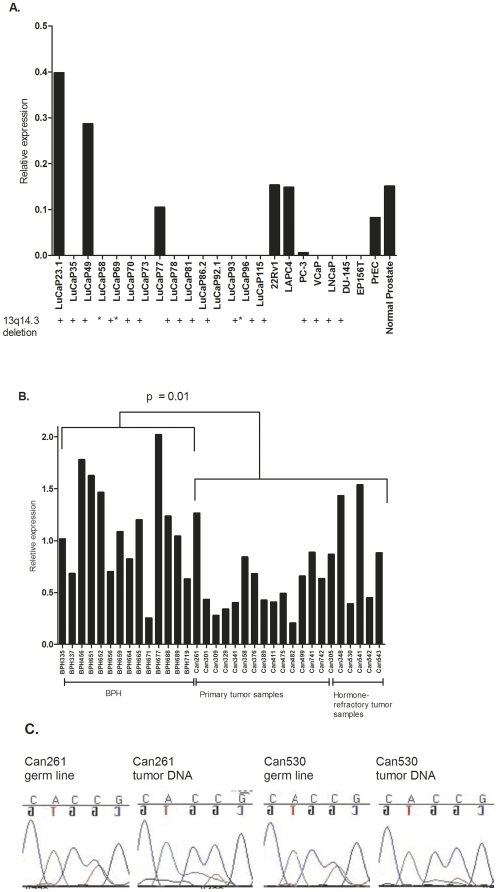
Relative expression of *ARLTS1* in A, LuCaP xenografts, prostate cancer cell lines, prostate epithelial cells and in normal prostate and B, in BPH and clinical tumor samples, as determined by Q-RT-PCR. The Cys148Arg (T442C) genotype as well as the choromosomal aberration is shown. **C**, Cys148Arg (T442C) variant in two clinical cancer tissue samples and in two normal blood DNA (sequences are in reverse orientation). * determined as in Saramäki OR et al., 2006.

*ARLTS1* expression levels were also analyzed in fifteen LuCaP xenograft samples, six PCa cell lines, two prostate epithelial cell lines and in RNA from normal prostate sample. Among the analyzed xenograft samples, a significant reduction in *ARLTS1* expression was observed in two of the samples (13%) when compared to its expression in normal prostate sample (Fig. 3A). Total loss of expression was seen in 11 (73%) of the xenograft samples (Fig. 3A). Two of the xenograft samples, LuCaP23.1 and LuCaP49, had higher *ARLTS1* expression levels compared to the normal tissue. Six of the fourteen (43%) *ARLTS1* down-regulated xenograft samples had a genotype CC for the Cys148Arg (T442C) variant. When array comparative genomic hybridization (aCGH) was performed using these samples, 12/15 xenograft samples showed a loss on the chromosomal region of 13q14.3 (Fig. 3A).

In PCa cell lines, 5/6 (83%) showed a decreased or lost expression of *ARLTS1* when compared to normal prostate RNA (Fig. 3A). An *ARLTS1* locus deletion at 13q14.3 was found in PC-3, VCaP, LNCaP and DU-145. Among these, the latter also had the CC genotype for Cys148Arg (T442C). Interestingly, the Cys148Arg (T442C) variant was the only *ARLTS1* variant found in any of the PCa cell lines. Prostate epithelial cell line EP156T showed loss of expression. These results likely indicate that *ARLTS1* expression is low in the PCa epithelial cells.

*ARLTS1* expression was also assessed with the same approach in a panel of fourteen BPH tissue samples, fourteen primary and six hormone-refractory tumor samples (Fig. 3B). *ARLTS1* expression was signinificantly lower (*P* = 0.01) in clinical tumors compared to BPH samples, which further supports its role as a tumor suppressor protein (Fig. 3B).

In clinical prostate tumors, the existence of LOH was studied. Germline DNA from patients carrying the Cys148Arg (T442C) variant was analyzed by direct sequencing. Sequencing results showed that in two cases (samples 261 and 530) the T allele in blood had been replaced with a C allele in cancer tissue (Fig. 3C) which suggests a role for *ARLTS1* in tumor suppression i.e., inactivation of both alleles in carcinogenesis. When clinical parameters (WHO grade, Gleason and T-scores) were examined, one of the two variant-carrying “LOH-patients” had a Gleason score of 9 and WHO grade of 3, indicating an aggressive form of the disease. The other case had a WHO grade of 3 but no Gleason score was available.

**Figure 4:**
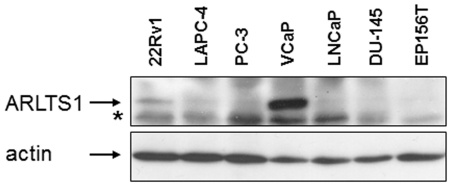
The levels of ARLTS1 protein by Western blotting. Protein expression status in six prostate cancer cell lines and in one normal prostate epithelial cell line. Actin is shown as a loading control. ARL11 expected/observed Mw 21.4 kDa. A non-specific band is marked with an asterisk (*).

ARLTS1 expression was further determined by Western blotting (Fig. 4). Cell lysates were available from six prostate cancer cell lines, (22Rv1, LAPC-4, PC-3, VCaP, LnCaP, DU-145) and from the normal prostate epithelial cell line EP156T. ARLTS1 expression was highly convergent to ARLTS1 mRNA status showed in Fig. 3A. As expected, cell lines LnCaP, DU-145 and EP156T showed negative expression. In prostate cancer cell lines 22Rv1 and PC-3 ARLTS1 expression was corresponding to relative mRNA expression values, whereas in PCA cell line LAPC4 protein expression status was lower than the mRNA expression level. PCA cell line VCaP showed the highest ARLTS1 expression which was not corresponding to negative relative expression status seen in Q-RT-PCR.

When clinical characteristics (age at diagnosis, PSA value at diagnosis, T, N, and M-stage, WHO grade and Gleason score) were included in the analyses of germline genotype data, the CC genotype was significantly associated with a younger age at diagnosis and PSA value (*P* = 0.05 and *P* = 0.03, respectively) among the unselected material. This phenomenon was also observed in our previous study where the CC genotype of Cys148Arg (T442C) was associated with high Gleason scores (*P* = 0.01) and high PSAs (≥7) at diagnosis (*P* = 0.05) [7]. When the correlation between aggressive disease status and the occurrence of the CC genotype was examined, we found a statistically significant association (*P* = 0.02) among the unselected cases (Table 3). Within familial PCa cases, no association was found between the CC genotype at the Cys148Arg (T442C) locus and any of the clinical variables or with aggressive PCa.

**Table 3:** Association of the Cys148Arg (T442C) variant with clinicopathologic variables of prostate cancer patients.

	Familial cases	Unselected cases
No. of subjects	212	1848
**Aggressive disease**		
No. of subjects	121	975
TT	40	281
TC	57	471
CC	24	223
OR (one-sided 95% CI), *P* value [CC] *vs.* [TT+TC]	0.62 (0.35 - infinity), 0.95	1.28 (1.05 - infinity), 0.02

*ARLTS1* expression in the different tissues of the body is shown in Figure S1. The figure illustrates that *ARLTS1* is highly expressed in the hematological and lymphatic system. The results from co-expression analysis illustrate a strong association with immune system processes, revealing an enrichment score for the cluster of 6.67 and a p-value of 2.1e-7. These results were generated from the top 100 genes with a correlation value greater than 0.3 among normal samples. Among cancer samples, the category of immune system processes had the highest enrichment score 14.89 with a p-value of 5.3e-21 and adjusted Benjamin score of 2.8e-17. We did not identify any strong gene ontology associated with the top 100 genes that were negatively correlated with *ARLTS1* among cancer samples. We confirmed our findings using a separate dataset from expo (IGC: Expression Project for Oncology. 2008 [http://www.intgen.org/expo.cfm]). The results illustrate that 56% of the genes were commonly identified to positively correlate with *ARLTS1* in either dataset comprised of cancer samples. The genes with a positive correlation above 0.3 identified in the intersection yielded a very strong gene ontology enrichment score of 18.12 for immune system process with a p-value of 2.1e-24 as shown in Figure S2. Gene expression among PCa specimens is shown to be very low for *ARLTS1*. We could not reliably establish any significant link to PCa for *ARLTS1* using gene expression data due to low expression levels.

## Discussion

The *ARLTS1* gene and *ARLTS1* polymorphisms have been shown to have a role in the pathogenesis of many cancers. The nonsense polymorphism at the end of the coding region has been revealed to predispose to familial cancer [8]. Functional analyses of the truncated protein have indicated that the Trp149Stop variant might affect apoptosis and tumor suppression. Another *ARLTS1* variant, the missense polymorphism Cys148Arg (T442C), and especially the CC genotype, has been found to be significantly associated with high-risk familial breast cancer [4]. The same variant was also found to be associated with predisposition to melanoma [5]. The roles of *ARLTS1* variants have also been studied with colorectal cancer [23] and chronic lymphocytic leukemia (CLL) [24] but no associations have been found.

In the present study, we showed an association with the *ARLTS1* Cys148Arg (T442C) variant and elevated PCa risk, validating our previous results [7]. Previously, we sequenced 164 familial and 377 unselected PCa samples together with 809 controls, and Cys148Arg (T442C) showed significant association with PCa risk (OR 1.19; 95% CI 1.02–1.39, *P* = 0.020) but the significance did not hold when the data were subdivided into familial and unselected cases. However, when the C allele was assumed to be recessive, all data (familial and unselected) showed significant association between the CC genotype and PCa risk (*P* = 0.005). Here were genotyped more familial and unselected PCa cases and Cys148Arg (T442C) revealed a statistically significant association with PCa risk among both familial (OR = 1.67, 95% CI = 1.08–2.56, *P* = 0.019) and unselected PCa patients (OR = 1.52, 95% CI = 1.18–1.97, *P* = 0.001). In the combined data set of both familial and unselected cases, the overall risk was even more increased (OR = 1.54, 95% CI = 1.20–1.98, *P* = 0.0007). The fact that previous analysis did not hold when divided to subclasses could therefore be explained by the size of the study. The current study has a power of approximately 100% to detect association compared to 94% of the previous study. Further follow-up with larger sample size would likely make the association even stronger. We found no association between this variant and BPH, suggesting that role of the Cys148Arg (T442C) is minor and the cancer predisposing effect emerges only in more advanced cases, especially those with an aggressive PCa status. Alternatively, BPH and PCa are independent events or at least *ARLTS1* is not consequential for BPH transformation to PCa. The longer follow-up of the BPH patients is needed to assess the question whether the patients carrying CC genotype (16%) are the ones developing cancer later on.

Only the homozygous form of the C allele of the Cys148Arg (T442C) variant resulted in an association with PCa risk when compared to the TT genotype. This supports the results from previous studies with breast cancer [4] that showed that C is the risk allele while the T allele has a protective or neutral effect on carcinogenesis. *ARLTS1* Cys148Arg (T442C) in particular has a predisposing effect on sporadic PCa. Further, the Cys148Arg was the only *ARLTS1* variant observed at a greater frequency among PCa tissue samples and xenografts, where the frequencies were 28.3% and over 40 percent, respectively. This also indicates a likely role for *ARLTS1* in prostate carcinogenesis. Interestingly, a total absence of Trp149Stop (G446C) was observed both in clinical tumor samples and in xenografts indicating that this variant has not an important role in PCa development.

The control population in our study was not in Hardy-Weinberg equilibrium (HWE), suggesting that Cys148Arg might be a novel variant. In our data, an excess of heterozygotes was observed which may indicate the presence of over dominant selection or the occurance of outbreeding. The phenomenon of heterozygous advantage allows for natural selection to maintain the polymorphism. The same control population has been used many times in different analyses, and never before has it been discordant from HWE [25]. Interestingly, in the Sub-Saharan African population, the CC genotype does not exist, and in the Asian population it is only present in a small fraction (http://www.ncbi.nlm.nih.gov). Only within the European population does CC exist at a greater proportion. Considering the possible role of *ARLTS1* in the immune system, it may be that the CC genotype has provided a protective effect among Europeans. HW disequilibrium among the controls is not either result of genotyping error because direct sequencing was previously used to genotype 164 of the familial PCa samples and the genotypes matched with the TaqMan assay results. Furthermore, the same assay was used to genotype all the cancer samples and controls.

Quantitative reverse-transcriptase-polymerase-chain-reaction assay (Q-RT-PCR) showed lowered *ARLTS1* expression in most of the clinical PCa samples, compared to BPH samples. This is consistent with the previous findings; *ARLTS1* expression has also been detected to be very low or absent in lung carcinomas and CLL cells [8], [26], as well as in ovarian primary tumors and cell lines [27], when compared with the levels of their normal counterparts. Here, using Q-RT-PCR, we used commercially available normal prostate RNA from a 72 year-old male. With respect to PCa disease progression, we could assume that at the age of 72 there might be some changes in the prostatic tissue, and use of control RNA from a younger male might lead to even better results concerning comparisons of *ARLTS1* expression status in normal and diseased samples.

Western analysis of prostate cancer cell lines for levels of ARLTS1 protein was in most part convergent with the ARLTS1 mRNA levels. However, in one sample with 13q14.3 deletion (VCaP), high protein expression was detected. This divergent result may originate from several reasons and needs further investigation. ARLTS1 tumor suppressor gene may have duplicated or activated pseudogenes, or there might also be pseudogenes or polymorphisms within the regulatory miRNAs. However, these protein expression results further confirm that ARLTS1 expression is low in the PCa epithelial cells.

The suggested mechanisms behind the reduced or absent *ARLTS1* expression are variable, including promoter hypermethylation or LOH. It has been confirmed that in lung cancer cell lines and in ovarian carcinomas *ARLTS1* is down-regulated due to DNA methylation in its promoter region [26], [27]. *ARLTS1* restoration by adenoviral transduction induced apoptosis. Finally, re-expression of *ARLTS1* suppressed ovarian and lung cancer tumorigenity in nude mice. We were able to show that LOH exists also in PCa samples, meaning that heterozygous deletions may affect *ARLTS1* expression levels. *ARLTS1* resides at 13q14.3, a region that has been reported to be deleted in a variety of hematopoietic and solid tumors [8], including PCa. Future investigations include whether reduced *ARLTS1* expression in PCa may be due to hypermethylation.

Chronic or recurrent inflammation has been implicated in the initiation and development of several human cancers, including those of the stomach, liver, colon, and urinary bladder and a role for chronic inflammation in the etiology of PCa has been proposed [28]. The source for prostatic inflammation is unclear, but it has been suggested that it may be directly related to prostate infecting agents (such as sexually transmitted organisms and viruses), dietary factors and oxidative stress, urine reflux, chemical and physical trauma or a combination of these. Recently experimental evidence was provided by Schlaberg R et al that xenotropic murine leukemia virus-related virus (XMRV) was present in malignant prostatic cells and was significantly associated with more aggressive tumors [29]. Susceptibility genes related to PCa might function in host immune responses and protection against cell and DNA damage caused by oxidative agents. For example, two suggested susceptibility risk genes for hereditary PCa, *RNASEL* and *MSR1*, are both involved with innate immunity. Lymphoblastoid cell lines represent an essential component of the immune response, so this result may indicate that ARLTS1 has a function in immune system processes.

Alterations in important molecular pathways involved in PCa have been implicated in proliferative inflammatory atrophy (PIA) and prostatic intraepithelial neoplasia (PIN) lesions. Tumor suppressor genes NKX3.1 [30], CDKN1B which encodes p27 [28], [31] and the phosphatase and tensin homologue (PTEN) [32] are highly expressed in normal prostate epithelium and have shown to be down-regulated or absent in PIN and PCa. Since ARLTS1 acts as a tumor suppressor protein and has a decreased expression in PCa, these recent findings are in line with the results from other suggested PCa tumor suppressor proteins.

Previously it has been shown that *ARLTS1* induces apoptosis in lung cancer cells [8], [26] and in ovarian carcinoma [27]. In the case of prostatic inflammation and antigen engagement, the need for apoptosis increases and the processes of the immune system, together with endogenous inflammatory cells (such as T and B lymphocytes) become activated. Here we showed that in the lymphoblastoid cell lines of PCa patients, *ARLTS1* expression was significantly decreased among the CC carrying patients compared to the wild-type allele T carrying patients. The lymphoblastoid cell lines were derived by Epstein-Barr virus transformation of peripheral mononuclear leucocytes from patients, indicating that these cells have encountered antigen stimuli via viral infection. The CC carrying patients had a low *ARLTS1* expression status suggesting that *ARLTS1* function is decreased due to this risk genotype and consequently this leads to decreased apoptosis. This may indicate that the Cys148Arg (T442C) CC genotype contributes to the immune response by diminishing apoptosis rates, decreasing defense mechanisms and finally cancer progression. It has been shown earlier that lung cancer cells carrying the Trp149Stop (G446A) variant and expressing the truncated protein had a reduced capability to induce apoptosis compared to cells expressing the full-length protein [8]. This reduced apoptosis could also be the causal factor in PCa. Our data suggest for the first time that the predisposing effect of the CC genotype of Cys148Arg (T442C) is related to reduced expression in immune system cells (lymphoblasts) rather than in tumor cells where *ARLTS1* expression is naturally very low.

Like all other organs, the normal prostate contains endogenous inflammatory cells and immune response mechanisms. Consequently, the function of *ARLTS1* may not exclusively be based on tumor suppression as suggested before, but also on immune response functions that occur either locally in prostate or in peripheral tissues. This is in line with the findings that PCa is a very heterogeneous disease and different mechanisms of cancer progression may occur.

Taken together, association and expression analyses of the Cys148Arg (T442C) CC genotype and PCa risk suggest that *ARLTS1* Cys148Arg (T442C) variant has a role in PCa predisposition and *ARLTS1* functions via immune system processes.

## Supporting Information

Figure S1 **Expression of** ***ARLTS1*** **in the publicly available GeneSapiens database.** The expression of *ARLTS1* is very low in both normal and cancer samples. In the prostate the expression of *ARLTS1* remains very low and no significant associations could be made using gene expression data.
(TIF)

Figure S2 **A print from the EASE association analysis.**
(TIF)

Table S1 **Clinicopatholgic variables of prostate cancer patients.**
(DOC)
