# Supplementary figures and images for: Contribution of ARLTS1 Cys148Arg (T442C) Variant with Prostate Cancer Risk and ARLTS1 Function in Prostate Cancer Cells
Source: PLoS One. 2011 Oct 20;6(10):e26595. doi: 10.1371/journal.pone.0026595 (PMC3197657; doi:10.1371/journal.pone.0026595)

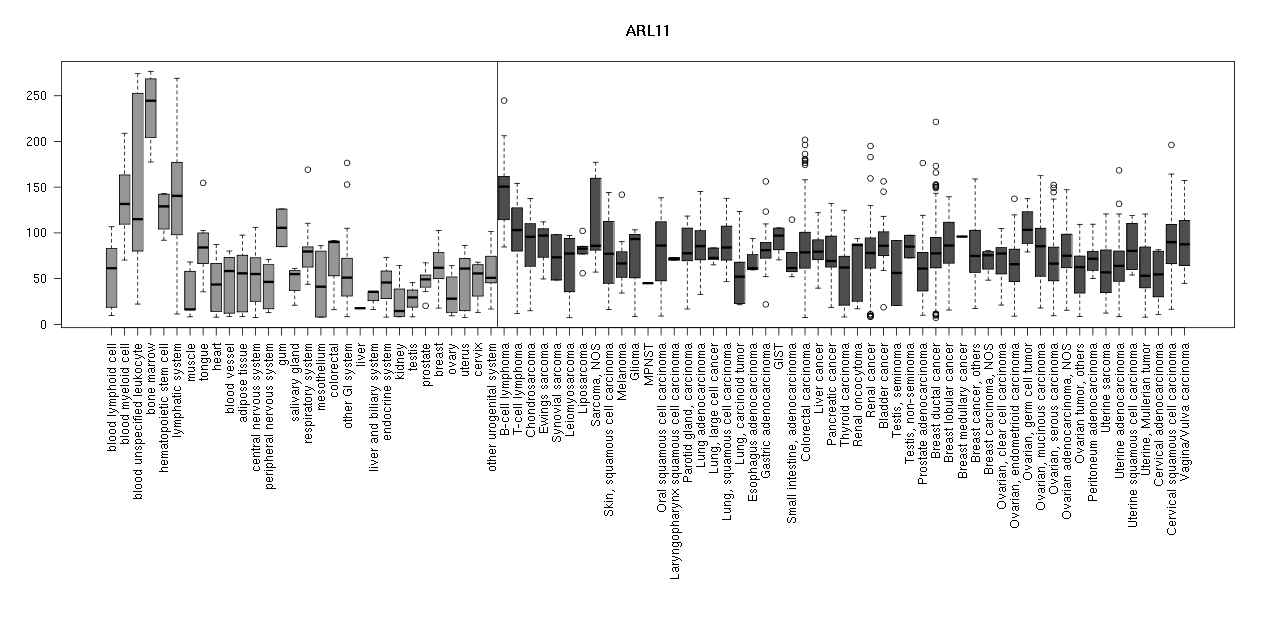

Supplement: Figure S1 — Expression of ARLTS1 in the publicly available GeneSapiens database. The expression of ARLTS1 is very low in both normal and cancer samples. In the prostate the expression of ARLTS1 remains very low and no significant associations could be made using gene expression data. (TIF) [file pone.0026595.s001.tif]

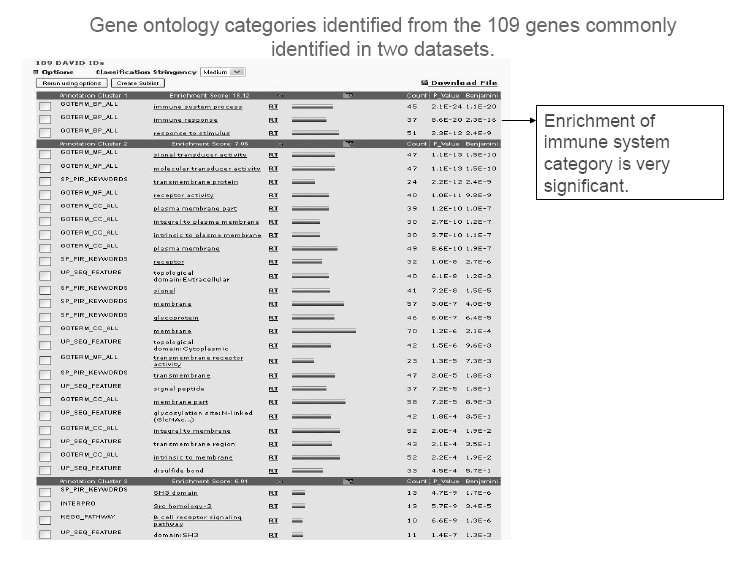

Supplement: Figure S2 — A print from the EASE association analysis. (TIF) [file pone.0026595.s002.tif]
