# Supplementary material for: Contribution of ARLTS1 Cys148Arg (T442C) Variant with Prostate Cancer Risk and ARLTS1 Function in Prostate Cancer Cells
Source: PLoS One. 2011 Oct 20;6(10):e26595. doi: 10.1371/journal.pone.0026595 (PMC3197657; doi:10.1371/journal.pone.0026595)
Supplement: Supplementary file 2 [file pone.0026595.s003.doc]

Supplementary Table 1. Clinicopathologic variables of prostate cancer patients

		Familial cases	Unselected cases
Characteristics	Variables	n (out of 181, %)	n (out of 1797, %)
Prostate specific antigen	< 4.0 ng/ml	10 (5.5)	166 (9.2)
	4.1 - 9.9 ng/ml	64 (35.4)	830 (46.2)
	10.0 - 19.9 ng/ml	41 (22.6)	403 (22.4)
	20.0 - 49.9 ng/ml	41 (22.6)	212 (11.8)
	50.0 - 99.9 ng/ml	11 (6.1)	79 (4.4)
	= 100 ng/ml	14 (7.7)	107 (6.0)
		n (out of 196, %)	n (out of 1806, %)
Tumor stage	T1	60 (30.6)	621 (34.4)
	T2	60 (30.6)	763 (42.2)
	T3	68 (34.7)	331 (18.4)
	T4	8 (4.1)	91 (5.1)
		n (out of 196, %)	n (out of 1806, %)
Nodal stage	N0	1 (0.5)	5 (0.3)
	N1	1 (0.5)	8 (0.4)
	NX	194 (99.0)	1793 (99.3)
		n (out of 195, %)	n (out of 1806, %)
Metastasis stage	M0	124 (63.6)	634 (35.1)
	M1	23 (11.8)	155 (8.6)
	MX	48 (24.6)	1017 (56.3)
		n (out of 118, %)	n (out of 1692, %)
Gleason score	2	0 (0)	11 (0.7)
	3	0 (0)	30 (1.8)
	4	1 (0.8)	49 (2.9)
	5	4 (3.4)	129 (7.6)
	6	49 (41.5)	665 (39.3)
	7	44 (37.3)	503 (29.7)
	8	12 (10.2)	143 (8.5)
	9	6 (5.1)	150 (8.9)
	10	2 (1.7)	12 (0.7)
		n (out of 118, %)	n (out of 1755, %)
WHO grade	1	17 (14.4)	433 (24.7)
	2	87 (73.7)	1068 (60.9)
	3	14 (11.9)	254 (14.5)
